# Movement Patterns and Use of Habitat Corridors in *Lacerta viridis* in a Semi‐Natural Habitat

**DOI:** 10.1002/ece3.71880

**Published:** 2025-09-11

**Authors:** Yoko Philipina Krenn, Harald Meimberg, Victor Sebastian Scharnhorst, Heimo Schedl

**Affiliations:** ^1^ Institute of Integrative Nature Conservation Research University of Natural Resources and Life Sciences Vienna Austria; ^2^ Institute of Landscape Development, Recreation and Conservation Planning University of Natural Resourches and Life Sciences Vienna Austria

**Keywords:** habitat network, Lacertidae, landscape connectivity, movement pattern, photographic re‐capture

## Abstract

Fragmentation of wildlife habitats by anthropogenic landscapes is a common cause of habitat degradation, highlighting the need to increase connectivity to support wildlife populations. This study focuses on the Eastern green lizard (*Lacerta viridis*) and investigates the effectiveness of a species‐specific established corridor network in allowing lizards to move between isolated habitat sections in a vineyard landscape. Using a non‐invasive photographic capture‐recapture approach, individual movements were tracked to assess sex‐specific patterns and distances covered in adult lizards. The study also provided insight into population parameters such as sex ratio and spatial distribution of the resident green lizard population. Results showed a surplus of male individuals and clustered distributions along the habitats. Both male and female green lizards use the habitat network, with males covering larger distances. The connectivity structures facilitated the movement of males between habitat sections, with some individuals covering distances exceeding 250 m. Hereby, male movement distances correlated with female abundance, which emphasizes the importance of connectivity structures in maintaining population stability. Two parameters correlated to female abundance showed significance in the models. First, the fewer females were detected in a male's core area, the higher the probability that it migrated a long distance. Secondly, a positive correlation existed between males' covered distance and female abundance in their entire activity range. Overall, this research highlights the importance of habitat connectivity measures for natural population dynamics by supporting male lizards to migrate in search of females.

## Graphical abstract

The study addresses the problem of habitat fragmentation for Eastern Green Lizards and evaluates the effectiveness of a species‐specific corridor network in a vineyard landscape. We have used a completely non‐invasive innovative photographic recapture method to track green lizards and assess individual movement patterns. Further analysis allows the assumption that males respond to the distribution of females in the study area.

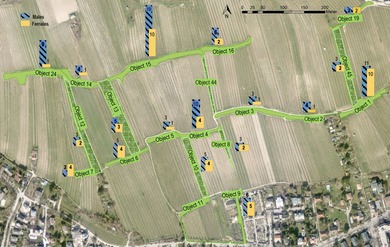

## 1 Introduction

The majority of European landscapes are characterized by fragmentation due to human activities. Consequently, natural habitats are extensively dispersed, with a significant number of species occupying semi‐natural, anthropogenically influenced habitats. (Bennett 2003; Sahlean et al. 2020). Biotope and habitat corridor networks are being established in various landscapes to connect separated habitat patches—either for one specific target species or as a multi‐species biotope network to allow species to migrate and disperse (Sahlean et al. 2020; Reck et al. 2023). Two types of connectivity can be mentioned in this context: Structural connectivity describes the potential connectivity between habitats through structures in the landscape, while functional connectivity captures the actual use of these structures by target species (Taylor et al. 2006; Singleton and McRae 2013). Habitat connectivity is typically assessed in presence‐absence studies over larger landscape areas to determine whether they are used by species (Braaker et al. 2014; Kor et al. 2022). Functional connectivity is considered to be a key element for the long‐term persistence of populations as it describes the actual usefulness of biotope corridors for species (Calabrese and Fagan 2004; Crooks and Sanjayan 2006; Singleton and McRae 2013; Beninde et al. 2016). According to the literature, connectivity structures should be determined and evaluated on a species‐specific basis (Beier and Noss 1998; Taylor et al. 2006). As shown in genetic studies of lizards in landscapes with connectivity elements, corridors are able to maintain gene flow between sub‐populations and fragmented populations in urban and other landscape types (Fink 2015; Orton et al. 2020). However, there is still a general lack of studies investigating the individual use of corridor elements instead of presence‐absence assessments, particularly for reptiles.

The Eastern green lizard (*Lacerta viridis*) is an endangered thermophile species found in Austria in climatically favorable habitats at the north‐western edge of its distribution range. This species is listed in Annex IV of the European Union's Flora‐Fauna‐Habitat Directive (FFH), which includes strictly protected animal and plant species of community interest (EU 1992).

Marginal populations of *L. viridis* in particular are considered to be potentially endangered as anthropogenic and natural influences can lead to the extinction of small populations. In fragmented landscapes, small populations are likely to be at higher risk of extinction, especially peripheral populations, due to lower genetic diversity compared to core areas of distribution. (Böhme, Schneeweiß, et al. 2007; Henle et al. 2017). It is assumed that edge populations are more sensitive to external influences and more dependent on microhabitat characteristics such as optimal radiation and slope (Prieto‐Ramirez et al. 2018). The main threats to the species are habitat loss due to human activities like habitat fragmentation and degradation, shrub encroachment, or land consolidation. In addition, open areas used as nesting sites appear to be limited (Schedl and Klepsch 2001; Sound and Veith 2001; Edgar et al. 2010). Moreover, species living in agricultural areas are generally at risk of exposure to pesticides. In Central Europe, up to 65% of the eastern green lizard's habitats could potentially be affected by pesticides as its climatically favored locations often overlap with typical wine‐growing areas (Mingo et al. 2016). In terms of mobility within an area, lizards appear to be highly dependent on protective structures such as vegetation that facilitate their use of corridors and similar habitat features (Zajitschek et al. 2012; Glandt 2018).

Here we study a population of *L. viridis* mainly inhabiting two parallel embankments in the “Nussberg” wine‐growing area in Vienna (Austria), between which habitat corridors have been implemented in the intensively cultivated vineyards to allow *L. viridis* to disperse. This system of habitat corridors has been established in cooperation with the winegrowers as a species‐specific conservation measure as part of a conservation project running from 2011 to 2013. This marked an important step in connecting the widely separated linear habitats of the green lizard within the intensively farmed vineyard area.

In this study, we investigated whether this habitat corridor system is used by green lizards and how the lizards move within the habitat area. This provided information about (1) the current population structure, such as approximate population size and distribution, (2) movement patterns and movement motivation of adult green lizards, and (3) utilization of the species‐specific habitat corridor network. Using an innovative approach with high‐resolution photography in the field, we minimized invasiveness and were able to track the movements of individuals within the study site over one active season. As most reptiles are secretive animals, it is important to audit a study site in an intensive survey with several observation days (Edgar et al. 2010).

We predict finding an intact population of lizards in the survey area that will allow us to infer population‐ and habitat‐related movement patterns. We expect that adult male lizards demonstrate a greater propensity for covering distances and engaging in exploratory behavior in comparison to their female conspecifics. Furthermore, it is predicted that individual‐ and population‐related factors will influence the distances covered by males. Due to the territorial behavior of this species (Elbing 2000; Grillitsch and Cabela 2001; Schedl and Klepsch 2001; Molnár et al. 2016), especially in males, younger (respectively smaller) males of the population possibly have to migrate between the territories of older ones to find females to mate with. We also hypothesize that lizards use the corridor network for migration and dispersal in the area between the two elongated main habitat embankments. As shown in previous studies, *L. viridis* is a relatively mobile reptile species that can cover quite big distances (Mikatova 2001; Molnár et al. 2016).

## 2 Materials and Methods

### 2.1 Study Species

With a length of up to 40 cm, *L. viridis* is one of the largest species of the lacertid family in Europe. Both males and females are green with a fine dark pattern on the body; older adults are brighter than younger ones, which also have brown shadings on the legs and tail. Adult males' heads have a blue coloring during the mating season in spring, while females do not change color. The scale patterns on the animals' heads are as individual as a human fingerprint and can be used to identify each animal individually throughout its life, as proven in previous studies that individually surveyed green lizards in their natural environment (Klepsch 1999; Elbing 2000; Schedl 2001).

The species is distributed mainly in south‐eastern Europe from the Balkans to the Black Sea region and can be found in central Europe in Hungary and parts of Austria, as well as in small relict populations in eastern Germany and the Czech Republic. The species is composed of several lineages as derived from mitochondrial haplotypes, some of which might deserve subspecies status, in particular an Adriatic lineage and a lineage in Greece and Turkey. (Böhme, Fritz, et al. 2007; Marzahn et al. 2016; Jauss et al. 2021). In Austria, the nominate form *L. viridis viridis* occurs, which represents the species in the northern parts of its distribution range.

In Central Europe, the eastern green lizard is mainly found in climatically suitable areas for viticulture, such as the Pannonian region and in warm, south‐facing Alpine valleys. In these regions, *L. viridis* mainly inhabits vineyards, ruderal sites, scrubland, and areas without vegetation as well as habitats with a forest‐steppe character (Grilltisch and Cabela 2001; Mikatova 2001; Pačuta et al. 2018; Sevianu et al. 2022). In Vienna, four separate populations of *L. viridis* are found at the north‐western edge of the city in vineyards and forest edges. There, green lizards inhabit mainly open areas, dry forest edges, shrubby grasslands with sufficient cover structures, or dry stone walls on arid, warm slopes with southern exposure along the Vienna Woods and the Danube region (Schedl and Klepsch 1999).

*Lacerta viridis* is a site‐related, territorial species that tends to stay in an individual home range where it spends most of its time, usually near structures used for basking or shelter, such as rocks or wood piles. During the mating season, males have a wide activity range to find receptive females to mate with (Klepsch 1999; Elbing 2000; Schedl 2001). Young males tend to be “floaters” between older males' territories, while older males usually remain in one territory and defend it against others (Molnár et al. 2016). The results of a study on the learning of lacertids showed that *L. viridis* has a higher spatial perception and a faster adaptability to new spatial situations than other related species. As a species of relatively complex, structured habitats, it appears to require well‐developed spatial perception skills, such as finding and remembering suitable shelters and other habitat structures in a fairly large home range (De Meester et al. 2022).

### 2.2 Study Area

**FIGURE 1:**
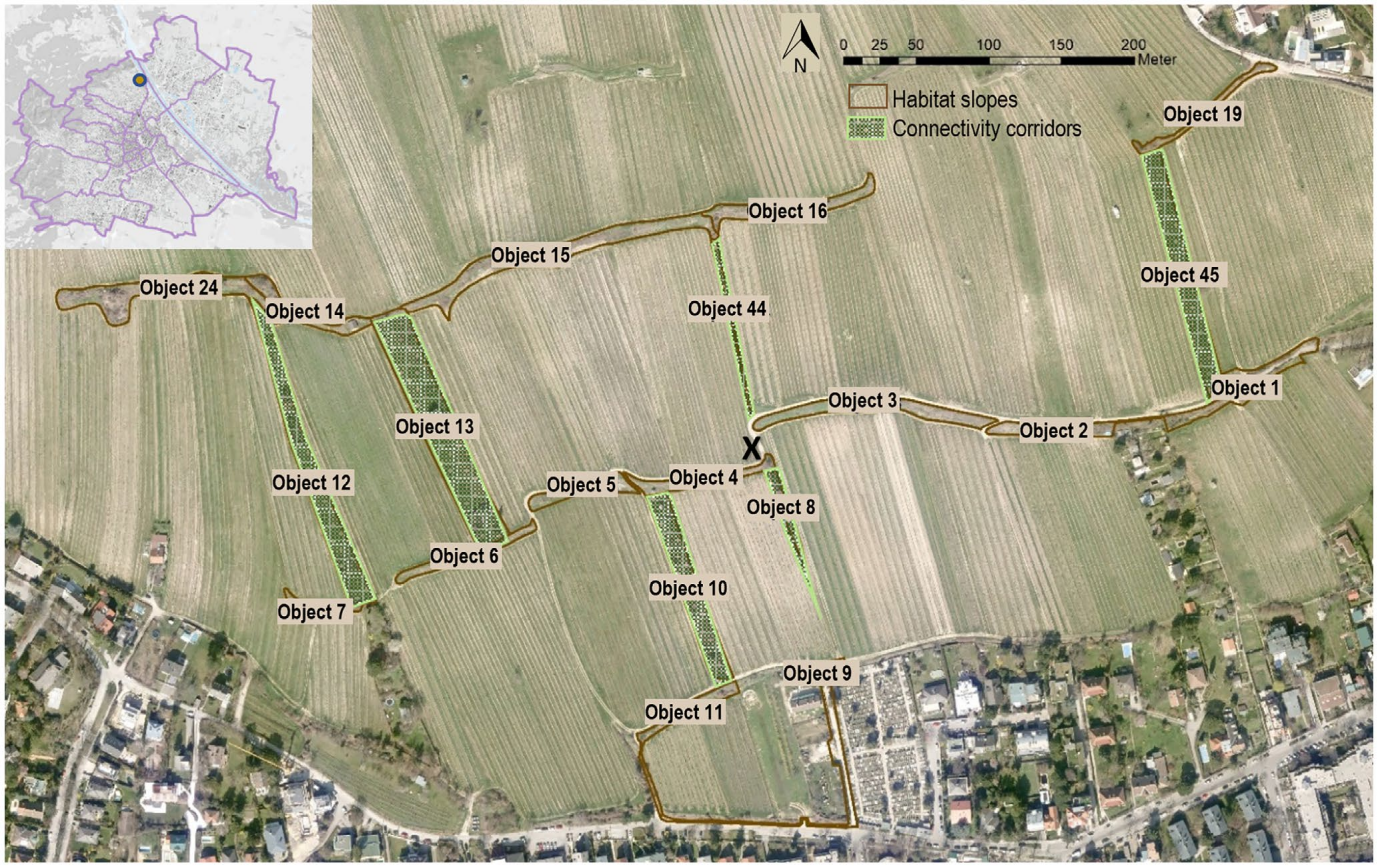
Study area: Vineyards of Nussberg, embedded habitat slopes (east–west) and orthogonal habitat corridors (north–south): Objects 8, 10, 12, 13, 44, 45 X ( 48.262762 N, 16.357017 E).

The study area is situated in the vineyard landscape on the Nussberg (48.262762 N, 16.357017 E), a south‐facing area of 23 ha in the north‐western region of Vienna, bordered by the Danube River and the hills of Leopoldsberg and Kahlenberg. The main lizard habitats are two parallel linear embankments at a distance of 130 to 200 m, with a total length of 1400 m (Figure 1). These habitat slopes are characterized mainly by herbaceous vegetation. Additionally, various structures such as rock and wood piles, clumps of shrubs, bushes, and dry‐stone walls can be found in certain areas. The six habitat corridors examined in this study span orthogonally between the main slopes, ranging in length from approximately 100 to 215 m. These corridors consist of narrow herbaceous semi‐natural structures, including unmown grass strips, rows of shrubs, and linear fallow areas. A total of 900 m of habitat corridors were surveyed and analyzed for this research. In previous projects, on which this study is based, the survey area was divided into fictitious sections to facilitate a simple geographical allocation of sightings (hereinafter called “objects” or “obj.”). Structures such as paths and tracks, as well as some natural features such as land use changes, etc., were used as fictitious boundaries between the object numbers.

### 2.3 Field Work

Surveys were conducted on 82 days between 4 March and 13 October 2017, between sunrise and approximately 1 h after sunset, from two to eleven hours per day, depending on weather conditions. Most of the sections were surveyed from the outside on linear paths. This was done in order to avoid invasive methods that could increase flight distances due to the high frequency of surveys, and also because the steep slopes were impassable (in many cases, this would have led to the destruction of habitats and their structures). We used a Sony DSC‐HX 400v bridge camera with 50× optical and 100× digital zoom, which gave the best results at a distance of about 3 m from the target. The survey paths were walked slowly and carefully to spot and photograph lizards before reaching their flight distance, ideally photographing both sides of the head without disrupting their natural behaviour. For each individual, we recorded the exact location and time, sex, age class, any injuries (such as tail autotomy), sun intensity and exposure, cover, behaviour, substrate, and habitat type. We estimated their age based on external parameters such as body and leg colouring, body size, and head size, and divided them into 3 age groups (2 years, 2–3 years, and 3 or more years). For an additional genetic study, 25 males were gently captured by noosing for buccal swabs and measurements. Snout‐vent lengths of these animals were included in statistical analysis (see below). It should be noted that females may be observed less frequently than males due to their secretive behaviour and better camouflage. This is taken into account when calculating the sex ratio, referred to in this study as the ‘observed sex ratio’. An approximation of the actual individual numbers was checked by plotting an accumulation curve of the first sighting times of all individual males and females.

### 2.4 Individual Identification

**FIGURE 2:**
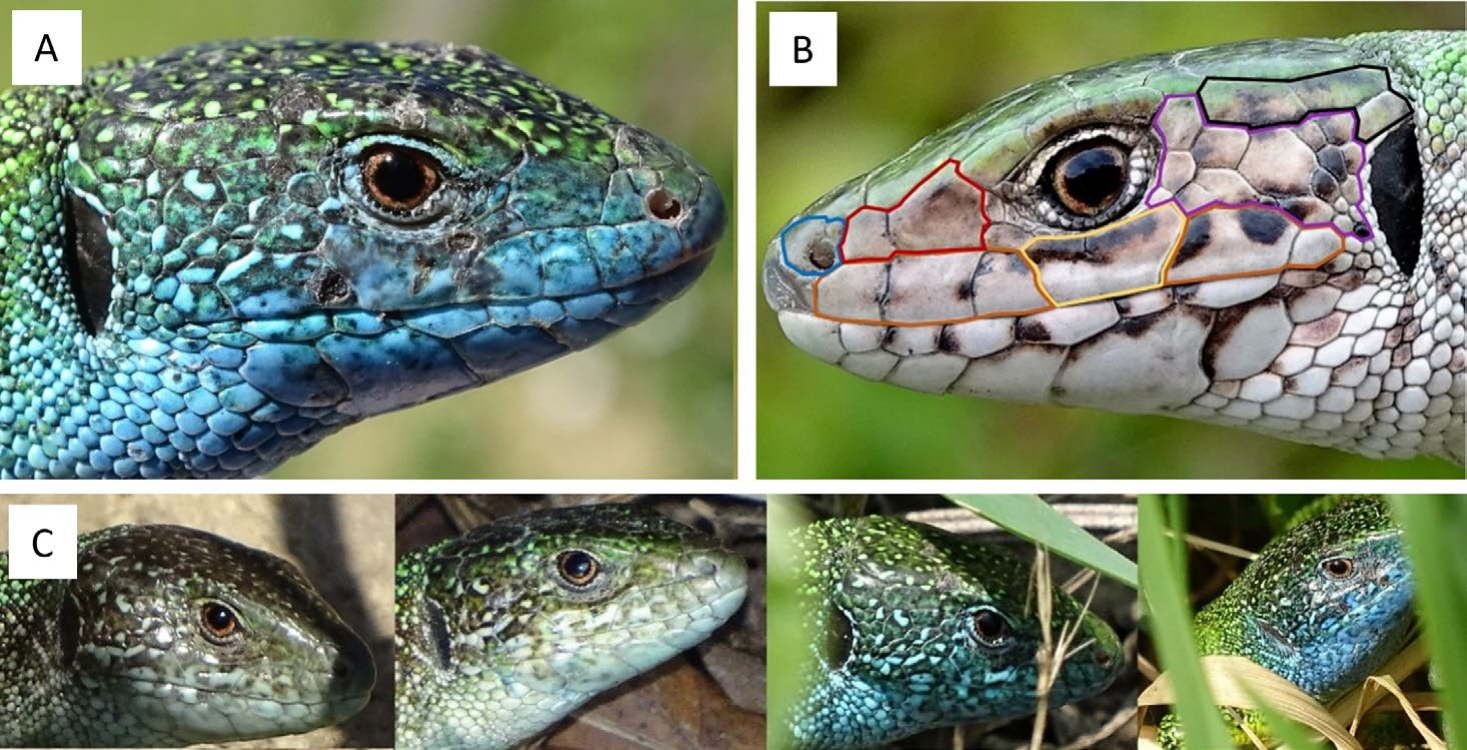
(A) Database image of the male 8 MB‐4MA‐5 MB showing optimal scale pattern detail (B) Individual recognition of the scale pattern on the head: Temporalia (purple), Supratemporalia (black), Labialia (orange), Subocular (yellow), Supranasalia (red), Nasalia (blue), shown on female (C) Photo set of male 8 MB‐4MA‐5 MB with four records between March and June.

Adult lizards can be individually identified by the pattern of scales on their heads (Elbing 2000; Steinicke et al. 2000; Schedl 2001; Sacchi et al. 2010). There are two clues to individual identification: the shape of the scales and the colour pattern within those scales (Figure 2). In particular, the shape of the scales, the connecting points between the scales, and the number of scales are individual for each specimen of *L. viridis* (Sacchi et al. 2010). In subadults, it is not possible to use the colour pattern within the scales for recognition, as the colours of the whole animal change until about two years of age, when it reaches its sexual maturity. Therefore, subadult lizards were not included in this study.

A photographic database was established for individual identification of *L. viridis* ; a picture was added to the database after it was cropped and rotated so that the final image showed only the horizontal side of the head (Figure 2). If both sides of the head were photographed, both images were included in the database. These photos were labeled according to a precise scheme that allowed the identification of details of the depicted animal. Once the database was completed, all photos were progressively compared to identify the animals, individually separated by sex. The photos of identified animals were collected in folders, and each individual was designated by a scheme that included a letter and the object sections in which the animal was recorded. Identification was performed manually due to the varying quality (angle and perspective) of the photos, which precluded the possibility of AI‐based analysis.

### 2.5 Data Processing and Recapture

**FIGURE 3:**
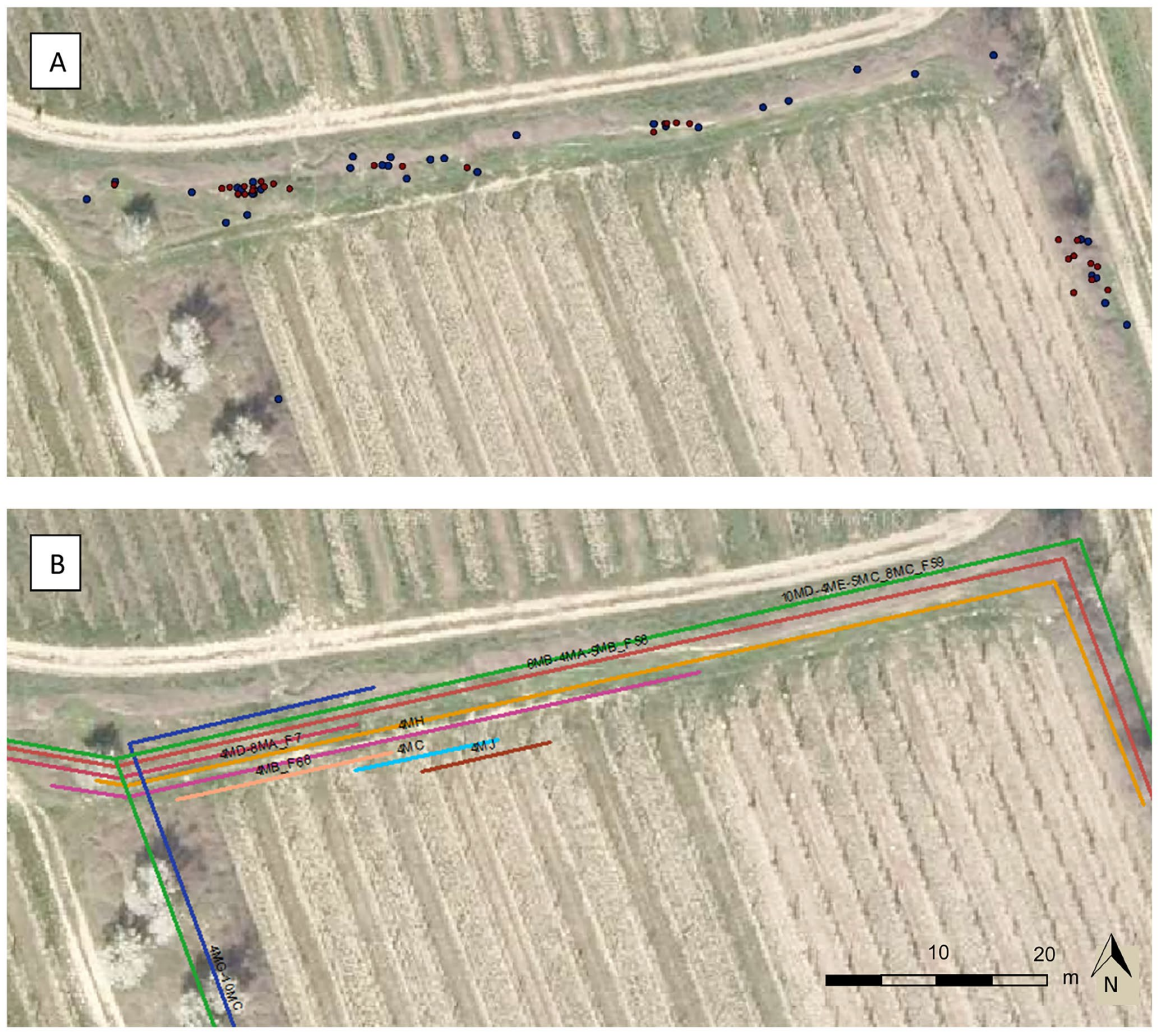
(A) Field record data points and (B) calculated distances of males, each line represents an individual's movement, projected parallel for better visibility, same map detail as figure (A).

The database containing records of green lizards was established in Microsoft Excel with all relevant data imported in chronological order. Additionally, the photo code system was implemented within the Excel database to virtually link the photos to their corresponding records. The geographical locations of all green lizards observed were subsequently transferred to ArcGIS 10.4.1 (ESRI 2016) for visualization and measurement of key parameters (Figure 3). To link the GIS‐map data points to the Excel database, the Join‐and‐Relate tool was used to include all relevant information (individual name, sex, date of record, structure used, etc.). Finally, it was possible to display all the data points for a particular individual in order to track their movements in the area. For each green lizard, the exact covered distance was measured in linear, parallel projected movement based on the linearly arranged habitats (Figure 3B). No animal was identified without a photograph in this study.

### 2.6 Statistical Analysis

**FIGURE 4:**
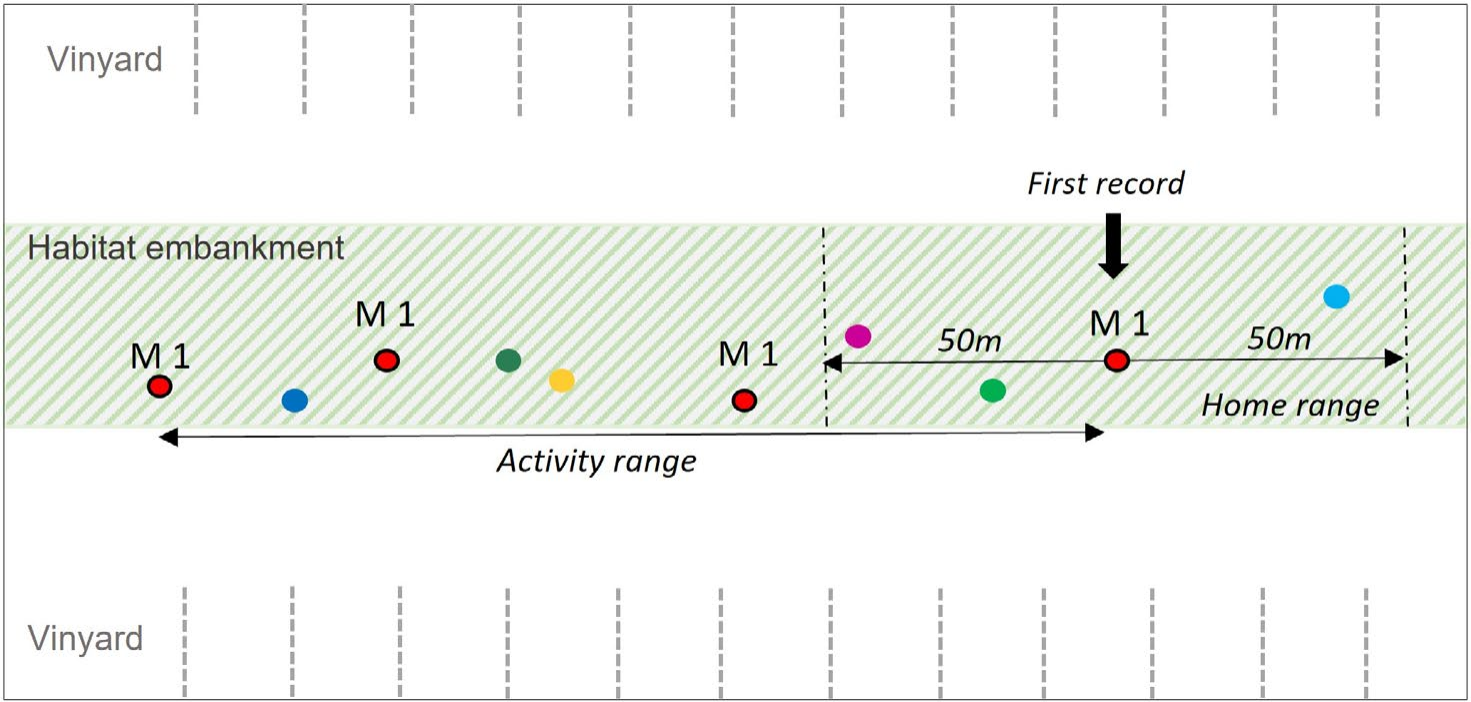
Scheme for GLM input: The right position of male 1 (M1) represents the point of the first record in the habitat embankment. The ‘home range’ is defined within a linear 50 m on either side of the point of the first record (100 m in total); the activity represents the total observed distance traveled. Additional points represent other lizards within the activity area and the home range of male 1.

All analyses were conducted in R (Version 4.2.2) using packages from the tidyverse and additional libraries (Wickham et al. 2019; R Core Team 2023). We used a generalized linear model (Gaussian‐distributed GLM with an identity link) to test how distances covered by males were influenced by the following predictors: (1) number of other males within a 100 m'home range, (2) number of other males within the total activity range (AR), (3) number of females within the home range, (4) number of females within the AR, and (5) approximate hatch year (categorised as 2014 or older, between 2014 and 2015, and 2015) and number of sightings. A male's *home range* was defined within a distance of 50 m from the first recorded location to both sides of the narrow embankment habitat and thus contained 100 m of habitat length, and its AR encompassed the entire observed distance that the individual covered in the survey period (Figure 4).

To account for uneven sampling effort, which potentially influences calculated distances, we included the number of sightings per individual male as an additional predictor. Because the response variable (distance covered) was not normally distributed, we applied a log transformation (natural logarithm) to the distance data in the GLM. Our model selection was guided by achieving the best balance between fit and interpretability. We selected the final model by comparing Akaike Information Criterion corrected for small sample sizes (AICc) employing the MuMIn package (Bartón 2025) and by using ANOVAs, then validated it with DHARMa diagnostic checks (Hartig 2022). A table of candidate models and relevant parameters, including AICc values and model weights, is provided in the supplements (Data S1).

The final model was:

$$\begin{array}{c} \log\;\left({\text{distance covered}}_{i}\right)=\beta_{0}+\beta_{1}\left(\text{hatch}\;\mathrm{yea}r_{i}\right)+\beta_{2}\;\left(n\;\text{females}\;100\;m_{i}\right)\\ \;+\beta_{3}\;\left(n\;\text{females}\;AR_{i}\right)+\beta_{4}\;\left(n\;\text{males}\;100\;m_{i}\right)\\ \;+\beta_{5}\;\left(n\;\text{males}\;AR_{i}\right)+\beta_{6}\;\left(n\;\text{record}s_{i}\right)+\varepsilon_{i} \end{array}$$

To clarify relationships among the predictor variables, we computed a matrix of Pearson correlation coefficients for all possible pairs using the Hmisc package (Harrel Jr. 2025). A correlogram is provided in the Supplements (Figure S1). Log‐transformed distance data and a subsequent ANOVA were also used to test for differences in distance covered between the sexes of *L. viridis* . Additionally, data from captured males (*n* = 24) were analysed via ANOVA to test the influence of snout‐vent length (SVL) on distance covered and to determine whether SVL differed significantly across hatch‐year categories. We used Tukey's post hoc tests (package emmeans, Lenth 2025) to compare hatch‐year groups for both GLM and ANOVA.

Statistical analysis was carried out only for distances covered by males.

## 3 Results

### 3.1 Population Data

**FIGURE 5:**
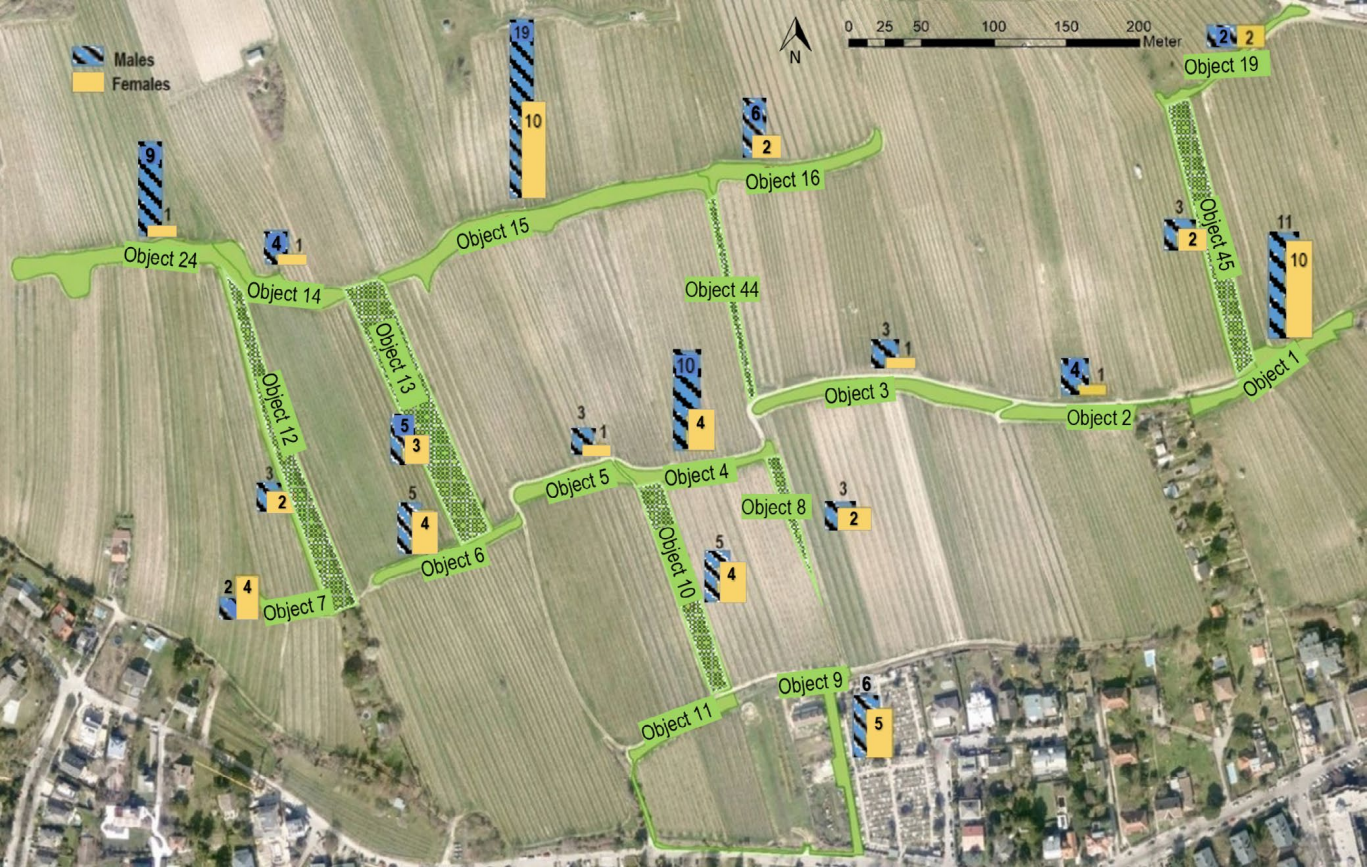
Distribution and sex ratio in the study area. Number of individuals: Males in blue crosshatched, females in yellow. The following figure includes all males and females per object number, so animals detected in more than one object are shown more than once in the illustration (Map data: Stadt Wien 2018).

Males and females of *L. viridis* were distributed differently across the study area, with males showing a more uniform distribution compared to females. In some sections of the study area, almost no females were detected, while in other parts, females were recorded more frequently (Figure 5). Habitats in the easternmost and upper parts of the survey area were densely populated, the latter showing an imbalance in the observed sex ratio of nearly 2:1 (m:f). Edge areas in the northeast and southwest showed a lower abundance of individuals, as did some parts of the central area. All habitat corridors (except obj. 44) showed the presence of adult males and females. The results of this study, in general, displayed a sex ratio of 1.5: 1 (m:f) in all detected lizards.

**FIGURE A1:**
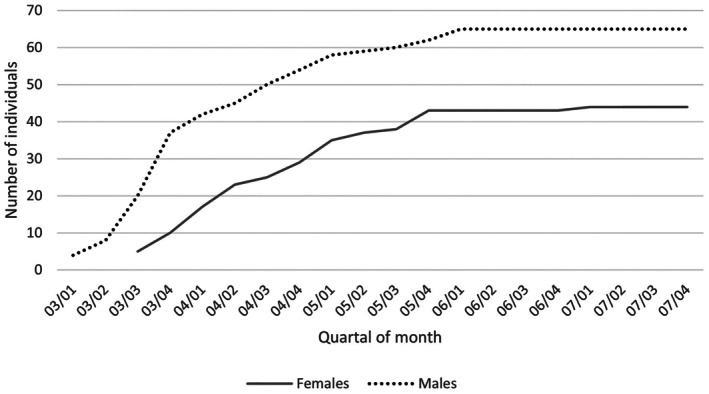
Accumulation curves of new recorded male and female individuals. Presented per quarter per month from March– July 2017.

The accumulation curves demonstrate a parallel rise in the number of new sightings of both males and females until the onset of June; here the data reveal a concurrent flattening of the curves among both sexes (see Figure A1).

Data from 454 photographed adult *L. viridis* were used to calculate movements and estimate population size in this study. In total, 540 adult green lizards were observed during field work; however, this number also includes animals that were not photographed due to flight, which therefore could not be included in the subsequent calculations (Table 1).

**TABLE 1:** Total number of records, adult animals photographed, and photo‐assigned.

Group	Records in total	Photographed animals	Assigned photos	Identified individuals
Adult males	376	335	322	77
Adult females	164	137	132	57

Of the 335 males photographed, 322 photos (96.1%) could be individually identified. Based on these images, 77 male individuals could be differentiated, corresponding to the minimum number of adult males present in the study area, which is referred to as the observed population size. On average, every male was documented photographically 4.9 times. For the 61 males that could be included in the calculation of migration distances because they were sighted at least twice, the mean record number was 5.1 ± 3.1 SD sightings.

A total of 137 females were photographically documented, of which 132 pictures (96.4%) could be individually assigned. Fifty‐seven females could be differentiated; this number also shows the minimum number of females present, with each female being observed 2.9 times on average. For the 29 females, which were recorded at least twice and therefore could be included in the calculation of migration distances, the mean record number was 3.6 ± 2.4 SD sightings.

In total, 98% of all photographed males and females could be assigned individually.

### 3.2 Movement Patterns and Motivation

**FIGURE 6:**
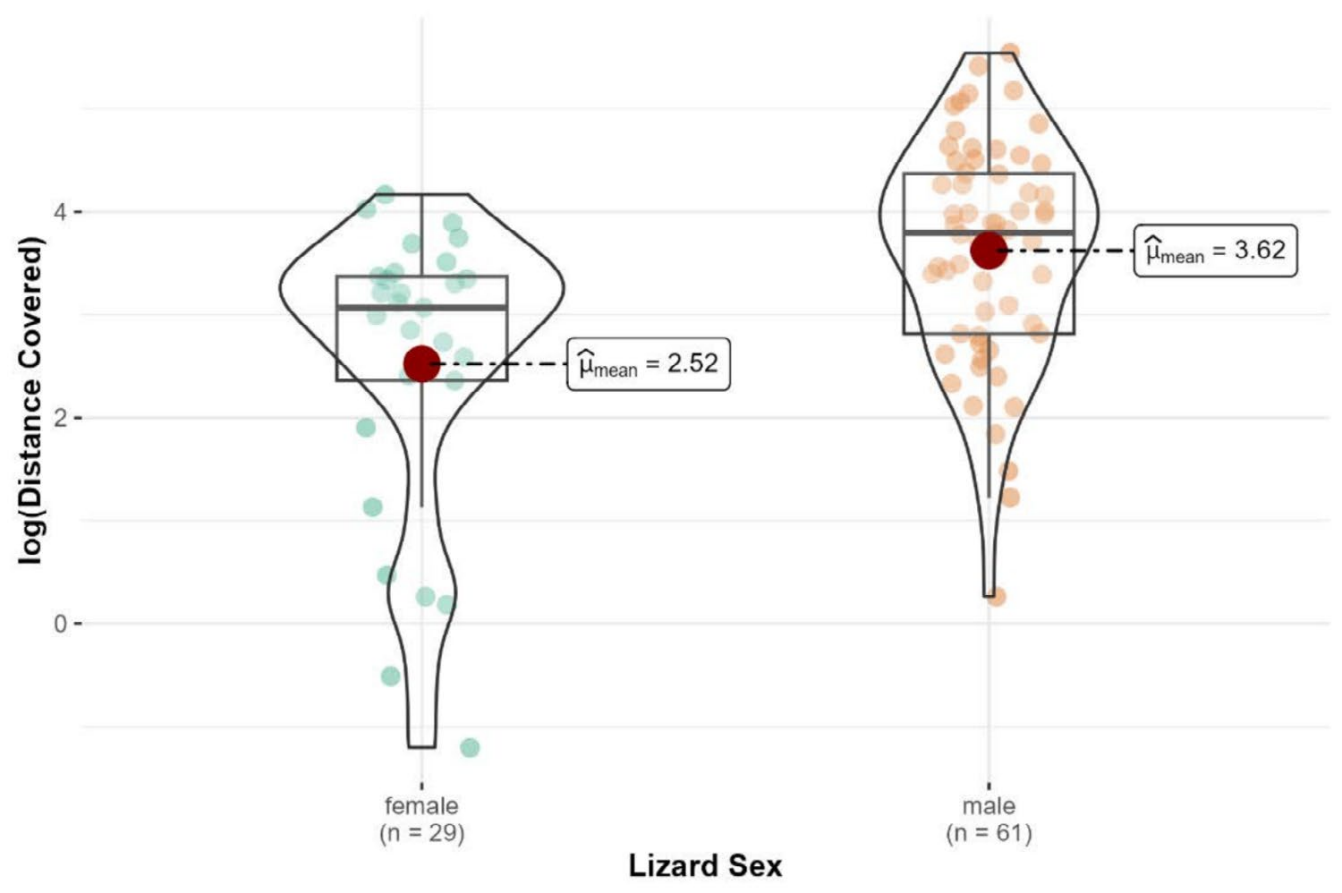
Comparison of the log‐transformed distance moved by male (*n* = 61) and female lizards (*n* = 29). Each point represents an individual lizard's distance (in meters, log scale), with the box outlines showing the interquartile range and the central line denoting the median, while the red dot denotes the mean value for each group. Whiskers extend to 1.5 times the interquartile range.

As shown by ANOVA analysis of log‐transformed distance data, males covered significantly greater distances than females during the observation period (*F*_1,88_ = 16.68, *p* < 0.001, Figure 6). The observed distances covered vary between individuals as well as between the sexes of *L. viridis* . Detailed distances covered by all individual males and females are given in Appendix 1.

Movements could be tracked for 61 adult males with an average covered distance of 59.1 ± 54.8 m (for detailed distances see Appendix 1). Twenty‐one recorded males used more than one object as a habitat. 53 (86.9%) of these males moved less than 99.9 m, while 8 (13.1%) males moved between 100 and 255.1 m. The minimum recorded distance for a male was 1.3 m, while the maximum recorded distance was achieved by a three‐year‐old male using three objects and covering a total distance of 255.1 m. Distances of 29 adult females were recorded, covering an average distance of 22.0 ± 17.0 m. The recorded distance for females varied between 0.3 and the maximum of 64.5 m (Table 2).

**TABLE 2:** Summary of lizards' movement and sightings: Mean distances and sighting numbers by sex, Mean ± SD of distances and mean ± SD of the sighting numbers by sex.

Sex	Number of individuals	Mean sightings (*n*)	SD sightings (*n*)	Mean distance moved (*m*)	SD distance moved (*m*)
Male	61	5.131	3.122	59.148	54.756
Female	29	3.552	2.369	21.976	16.969

For the 61 tracked males, a generalised linear model (GLM) revealed that hatch‐year category, the number of females in both the 100 m home range and along the total distance covered, which is referred to below as the activity range (AR), and the number of records significantly influenced the log‐transformed distance covered (Table 3, detailed data Appendix 2). Compared to the reference hatch‐year group (2014), males from 2015 covered significantly shorter distances (*p* = 0.028), whereas males from 2014 to 2015 showed no difference from the 2014 group (*p* = 0.884). The number of females within a 100 m home range had a strong negative effect (*p* < 0.001), whereas the number of females in the AR had a positive effect (*p* < 0.001). Sampling effort (number of records per male) was also positively associated with distance moved (*p* = 0.031). Although the number of males in the 100 m home range showed a positive trend (*p* = 0.063), neither this factor nor the number of males in the AR (*p* = 0.208) was statistically significant. Post hoc contrasts (Tukey‐adjusted) suggested that pairwise differences in movement among hatch‐year categories, while still showing trends for shorter distances covered in the 2015 cohort compared to both other groups, did not remain significant at the *α* = 0.05 level (all *p* > 0.05), likely reflecting reduced power after multiple‐comparison adjustments (Table S1).

**TABLE 3:** GLM data of distances covered: female number in the home range and female number in the activity area showed a significant influence on the distances traveled by males. Also, age (hatch year) and sampling effort (number of sightings) showed influence.

	Estimate	SE	Statistic	*p*	Sign.
(Intercept)	3.188	0.235	13.545	< 0.001	
Hatch year2014‐2015	0.026	0.178	0.146	0.884	
Hatch year2015	−0.453	0.201	−2.256	0.028	*
n_females_HR	−0.276	0.043	−6.353	< 0.001	***
n_females_AR	0.47	0.064	7.361	< 0.001	***
n_males_HR	0.078	0.041	1.897	0.063	
n_males_AR	−0.064	0.05	−1.275	0.208	
n_records	0.061	0.028	2.222	0.031	*

*Note:* Significances *= *p* < 0.05, **= < 0.01, ***p=0.001).

ANOVAs showed no relationship between snout–vent length (SVL) and distance moved (*F*_1,23_ = 0.013, *p* = 0.91). By contrast, SVL differed significantly among hatch‐year categories (*F*_2,22_ = 11.07, *p* < 0.001), with post hoc tests (Tukey‐adjusted) indicating that males from 2014 were larger than those from both 2014 to 2015 (*p* = 0.045, Table S2) and 2015 (*p* < 0.001), whereas these latter two groups did not differ from each other (*p* = 0.57).

### 3.3 Habitat Corridor Network

**FIGURE 7:**
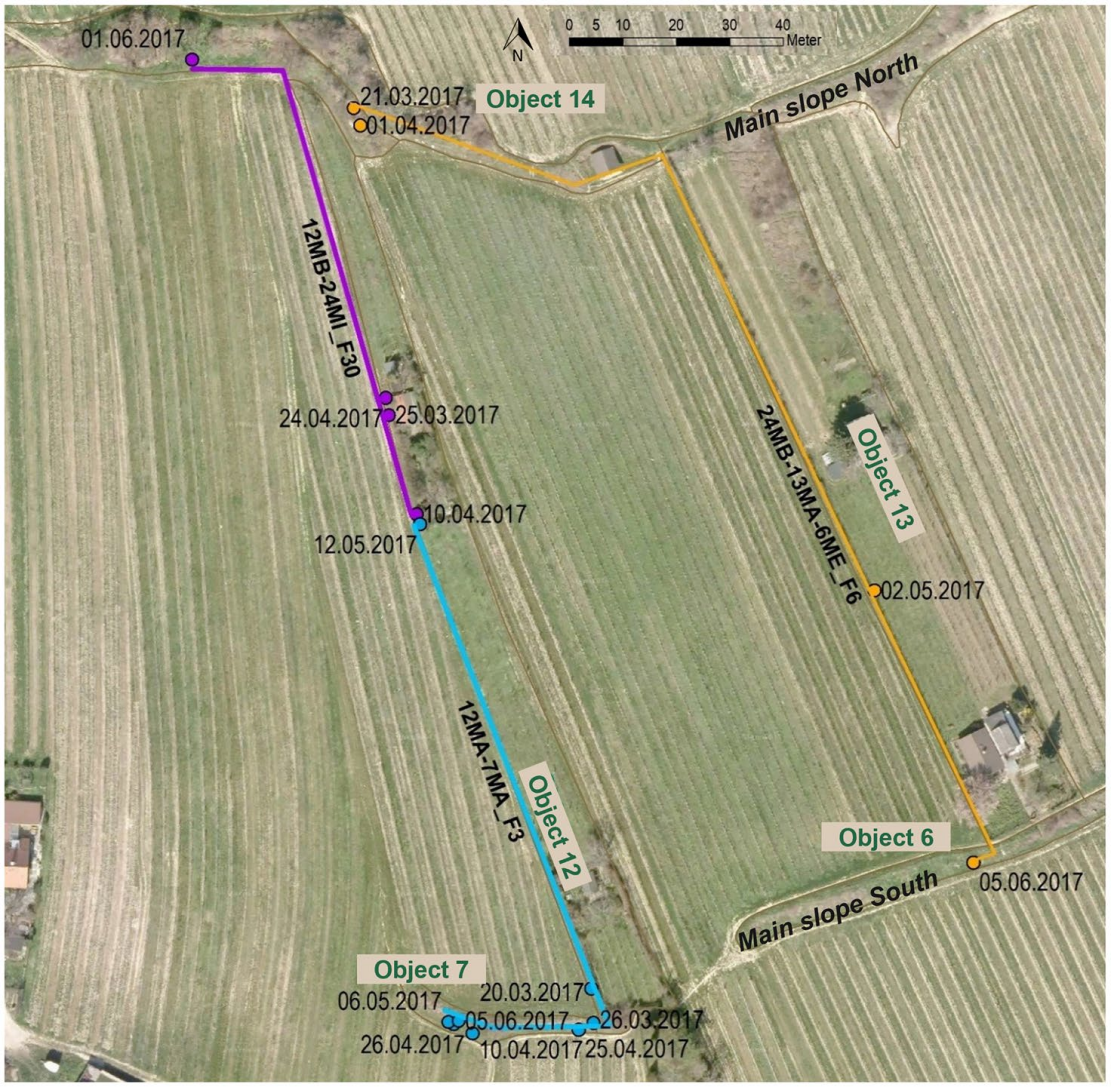
Example of movements in connectivity structures between the northern and southern main slope in the object. 12 and 13. Orange, purple, and blue represent the covered distances by three different males, including the record dates. One 3‐year‐old male (orange) migrated more than 255 m from the northern to the southern main slope using a habitat corridor object. 13, which is a narrow, unmown grass strip along a fence (orange). On May 2, it was found paired with a female in a corridor object. 13, and on June 5, in the lower main slope, close to a female as well. The neighbouring corridor was also used by two males: A 3‐year‐old male (purple) moved from the middle part of the slope, where it was first found at the end of March, and mated with a female (12WA) in a garden hedge in mid‐April, to obj. 24 in the upper main slope using a narrow grassy connecting structure with a length of about 100 m. The second male (blue) moved from the lower main slope object. 7 to the garden hedge in the central part of the connectivity structure, migrating in a small grass strip along an extensively cultivated vineyard in a private garden.

**FIGURE A2:**
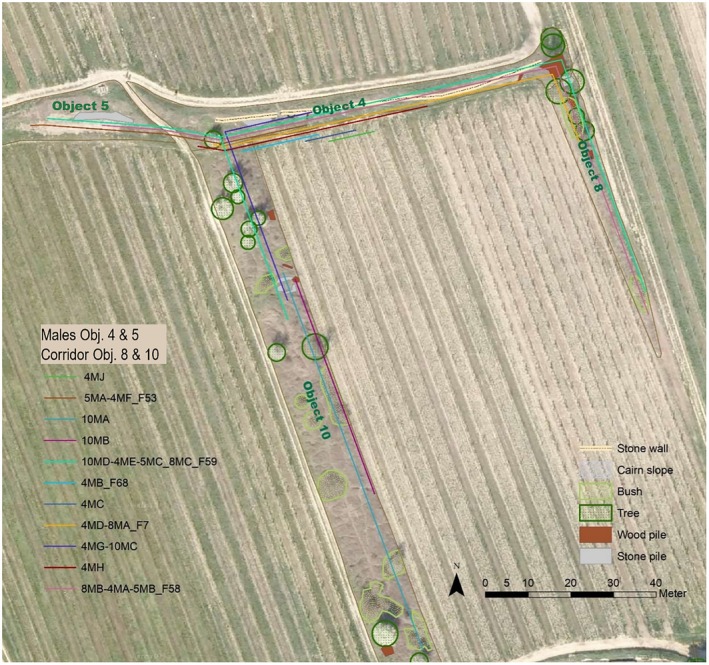
Males in objects 4 and 5 and in habitat corridors objects 8 and 10 (all distances of the observation period, arranged parallel for better visibility and clarity).

Green lizards using the connectivity corridors were observed in various areas of the study site. In the central area, males were observed in corridors obj. 8 and 10; in the western area, corridors obj. 12 and 13 showed the presence of males and females, as well as object 45 in the eastern part of the survey area (see Figure 7, Figure A2). Within these structures, movements were only recorded in males, who tended to cover more or less large distances in the corridor structures, whereas females were only observed at local points. One male observed covered 255 m using both the south and north main slopes and the corridor structure connecting the two main slopes for a distance of approximately 170 m.

Overlapping activity ranges were found around well‐structured sections like stone piles or walls with a connection to dense vegetation. Every male detected within a corridor was recorded in the habitat slopes adjacent to the corridor, used. Females were also only recorded in corridors, but most were only recorded locally, and none of these females were recorded in an adjacent habitat embankment as well (detailed numbers of animals within the different areas see Figure S2).

An example of reconstructed movements within an object and the use of connectivity structures is shown in the central part of the survey area (Figure A2). Activity ranges of males in obj. 4 are clustered around stone and wood piles in front of a south‐facing stone wall. Overlapping activity areas of up to eight males were recorded around the stone pile. Some males were found only around the cluster; others covered greater distances and also used corridors obj. 8 and obj. 10. In the central survey area, a young adult male (hatch year 2015) was recorded in 2 adjacent sections and the two associated habitat corridors covering a total distance of about 225 m in length. This male was recorded in the corridor structures, objects 8 and 10, and in the main slope, objects 4 and 5.

In the western part of the survey area, in corridors objects on 12 and 13, both males and females were observed. Males were found to move and reside in different parts of the corridors and neighboring habitat embankments, while females were recorded staying in a very small radius around specific structures like a fence or woodpile in the corridors.

In Figure 7, three examples of male movements within corridors. 12 and obj. 13 are given to demonstrate the migration of males between the northern and southern main slopes.

## 4 Discussion

### 4.1 Movement Patterns

*Lacerta viridis* is a mobile species, which is able to cover large distances in its habitat, correlating with the phenological and diurnal needs (Mikatova 2001; Molnár et al. 2016). Males showed distinct movement patterns compared to females, both in terms of distances and distribution within the study area. In lizard species, differences in movement patterns between males and females are generally observed, with males tending to cover larger activity ranges (Elbing 2000; Perry and Garland 2002; Schofield et al. 2012; Ryberg et al. 2019; Michelangeli et al. 2022). The results of our study confirm this also for the green lizard. In the Nussberg area, average distances of 60 m for males and 22 m for females were recorded, with maximum values of 255 m for males and 65 m for females, while the observed variance was large between the individuals. Additionally, we observed differences in the number of sightings: Females were sighted with an average of 2.9 records less frequently than males, while males were sighted on average 4.9 times. Comparable resighting differences between the sexes were found in a study of wall lizards (Abalos et al. 2020). In general, male green lizards are easier to spot in their natural habitats than females because the latter are much more secretive in their behavior and better camouflaged (Elbing 2000; Grilltisch and Cabela 2001). This factor may also account for the observed sex ratio of 1:0.74 (m:f); hence, other population studies found more balanced sex ratios in lizards (Elbing 2000; Schedl 2001). However, the accumulation curves of the newly recorded individuals show that the curve flattens out sharply around the beginning of June for both males and females, indicating that the detection rates were at a similar level for both sexes and therefore accurate. There is some unconfirmed evidence that the imbalance in the sex ratio may also be linked to the use of pesticides in the habitat (Mingo et al. 2017), which could definitely play a role in the Nussberg area, as most of the vineyards are cultivated conventionally.

Statistical analyses were performed on the movements of 61 adult males to find evidence of a relationship between population‐based parameters and distances covered. To determine if the sampling effort (sighting number) of males also showed influence in the model, we also included that factor. Sampling effort was slightly positively correlated with distance traveled, as we observed greater distances for males that we detected more often. This may also be an indication of how to successfully collect data on the distribution and conservation requirements of lizards.

The generalized linear model revealed a significant influence of the two female‐related parameters:

1. A negative correlation was found between the distances travelled by males and the density of females at the male's point of origin, i.e., the fewer females a male found at its home base, the more likely it was to travel greater distances.
2. There was a positive correlation between the distance travelled by males and the density of females throughout their range. This indicates that males who migrated greater distances generally had more access to females. According to this model, it can be assumed that males respond to the distribution of females.

Additionally, age showed an additional significant effect on covered distance.

In young males (hatched in 2015) we observed significantly shorter distances than in older males. A study on the influence of throat brightness and SVL on the spatial utilization strategies of *L. viridis* yielded similar results, with SVL showing a positive correlation with the territory size of male lizards in one study site. In contrast, another study site exhibited more random movement of males, without the statistical influence of those predictors (Molnár et al. 2016). Considering the general correlation between SVL and year of hatching in this species, these indicators could be used as predictors of the tendency to cover shorter or longer distances in territorial populations. However, contrary to our expectations, in our study population, younger males covered shorter distances than older males, while SVL alone showed no statistical influence. It has been observed that lizard males disperse to find females, which stay in smaller home ranges, where males are likely to “visit” them for mating (Ebrahimi et al. 2014). In other populations of green lizards, territorial males gather females around them, while floater males attempt to mate with ‘unguarded’ females in the same area. In a population with a surplus of females, males were observed moving back and forth between the females over long distances of more than 100 m to mate (Elbing 2000, 2016; Rehák 2015). Due to the low density of females in most sections of Nussberg, males would not benefit from territoriality and occupying an area if no or only a few females are present. Therefore, males from sections with few females are expected to cover greater distances to find mating partners in this study area. We observed one male from a section with very low female density migrating a long distance to meet females in other parts of the area, where it was found repeatedly in a corridor and another habitat bank paired with different females. In contrast, a younger male was observed in a section with a 1:1 male/female density, which was repeatedly found at approximately the same location paired with four different females in the mating season. Both examples of movement patterns can constitute successful strategies to produce offspring in a population with inhomogeneous female occurrence. Since we found the greatest distances covered by males during the mating season between April and June, we assume that this is the period of highest activity. There is evidence that habitat quality also has an influence on the movement patterns of lizards, as females tend to occupy high‐quality habitats, while only competitive males can stay in these areas due to strong competition between males (Abalos et al. 2020). Despite mating behavior, there obviously exist more reasons for movement in an animal's life, like predator presence, diurnal needs such as basking and feeding, or annual needs such as hibernation (Edgar et al. 2010; Glandt 2018). A Czech study reports that a green lizard population in Moravia even migrates over distances of 500–800 m between hibernation and summer activity sites (Mikatova 2001). On Nussberg, we assumed that the lizards hibernated close to the first record locations in March, as they were found close to stone walls or huge wood piles, sometimes covered with soil residue. Predators like the Kestrel (*Falco tinnunculus*) or the Smooth Snake ( *Coronella austriaca* ) were frequently observed in various sections of the whole study area.

For the observation of movement patterns, literature mainly contains spatial calculations based on the minimum convex polygon method or radio telemetry to determine the activity areas of lizards (Schedl 2001; Sound and Veith 2001; Sound 2005; Molnár et al. 2016; Ryberg et al. 2019). Due to the linear habitats present on the Nussberg, we applied linear distance calculations. Linear data was collected in another study area in Vienna, which showed mean distances of 180 m for males and 54 m for females along a vineyard edge on the Kahlenberg, with maximum distances of 459 m for males and 68 m for females (Klepsch 1999). These values reflect similar results to those of the current study on Nussberg, although we recorded lower maximum distances for males. Thus, *L. viridis* is a mobile species with high spatial requirements, which implies a well‐structured, interconnected network of habitats, which, however, do not necessarily require a large spatial extent, as narrow linear (edge) habitats are also utilized. There is evidence that *L. viridis* requires a patchwork of cover and shelter structures within scattered open areas in order to use a habitat to its full extent (Elbing 2000; Mikatova 2001; Pačuta et al. 2018). On Nussberg, due to the linearly arranged embankments with different kinds of vegetation, a quite varied and well‐structured patchwork of edge habitats is generally provided. Especially, interfaces between habitat types, such as open areas and denser vegetation or forest‐like edges, play an important role given that they provide a range of microhabitats and microclimates (Edgar et al. 2010). Individual personality traits may also play a role in exploratory courage, as there appear to be more exploratory and shy individuals in lizard species, which could also lead to distinct movement patterns in different individuals (Pačuta et al. 2018; Michelangeli et al. 2022). Fragmentation in the form of intensive vineyards, often without much ground vegetation, can also contribute to the loss of functional connectivity between the two surveyed embankments, which underlines the importance of connectivity corridors.

### 4.2 Habitat Corridor Network

In habitat corridors, primarily males were found using corridor sections for covering distances between the two main slopes. Females were found during mating season within these structures too, but mainly remained in a small area and did not cover any larger observed distances. The illustrated example of three males moving within two habitat corridors shows that the animals accept both grassy strips as well as woody or other denser vegetation for their movements. All three males were paired with females within the corridor structures during the mating season; however, almost no movements of the females could be recorded in these habitat parts as they moved in a very small range of less than 1 m. One female was found four times in the same location where it was recorded in May in a grassy corridor structure (obj. 13) along a fence, usually paired with a male. It is reasonable to assume that well‐structured corridors provide both the opportunity for migration and habitat for needs such as feeding, mating, basking, and hiding, as mentioned in the literature (Glandt 2018; Ersoy et al. 2019). Another corridor (obj. 10) also appeared to be a well‐accepted structure for migration: a 10‐m‐wide strip of fallow land that offered very heterogeneous structures for basking, hiding, and covering. Hedges and edge biotopes are also recommended as migration corridors as well as strips along paths, fallow land, and similar land covers to connect established biotope islands. The usefulness of corridors is particularly emphasized for migratory individuals, such as juveniles or floaters within a population, which are especially important individuals for small, dispersed populations as they are likely to explore and colonize new habitat patches and therefore may link sub‐populations within a meta‐population system (Edgar et al. 2010; Glandt 2018). At Nussberg, some of the males observed showed pronounced exploratory behavior as they moved long distances on the main slopes and in the connectivity corridors. It is reasonable to assume that they were searching for female partners, given that the main moving activity was observed in the months of the mating season. Only one of the six surveyed corridors showed no presence of lizards, a narrow and in some parts interrupted grassy strip. Maybe the patchiness of vegetation did not provide enough cover, which is a very important requirement for lizards and allows them to move safely beyond vegetation (Zajitschek et al. 2012; Glandt 2018; Ersoy et al. 2019; Taylor et al. 2021).

In a meta‐study, which investigated the benefits of corridor structures for different species, both species‐specific studies as well as those which only counted different species and recorded their presence in corridors, a notable fact mentioned was that corridors almost certainly support the movements of many species, not just the target species (Beier and Noss 1998). Generally, literature suggests that connectivity structures, and therefore functional connectivity, should be assessed on a species‐specific basis in order to obtain consistent results (Beier and Noss 1998; Crooks and Sanjayan 2006; Taylor et al. 2006). This study shows how a species‐specific corridor network is successfully used by the target species *L. virdis* for different diurnal needs in its life cycle.

## 5 Conclusion and Conservation

The current study provided insight into sex‐specific movement patterns and investigated the acceptance of a species‐specific habitat corridor network for eastern green lizards. Both males and females were recorded using the corridors, with males traveling greater distances using these structures to move between larger habitat patches. This demonstrated the usefulness of a corridor network between larger habitat patches for *L. viridis* and highlighted the importance of migratory structures in fragmented habitats. It was shown that vegetation strips and hedges can be successfully incorporated into a biotope network to allow eastern green lizards to disperse.

## Author Contributions

**Yoko Philipina Krenn:** conceptualization (equal), formal analysis (equal), methodology (supporting), visualization (equal), writing – original draft (lead), writing – review and editing (equal). **Harald Meimberg:** conceptualization (equal), methodology (equal), writing – review and editing (equal). **Victor Sebastian Scharnhorst:** formal analysis (equal), visualization (equal), writing – review and editing (equal). **Heimo Schedl:** conceptualization (equal), investigation (equal), methodology (equal), resources (lead), writing – review and editing (equal).

## Conflicts of Interest

The authors declare no conflicts of interest.

## Supporting information


**Figure S1:** ece371880‐sup‐0001‐FigureS1.pdf.

**Figure S2:** ece371880‐sup‐0002‐FigureS2.pdf.

**Table S1:** ece371880‐sup‐0003‐TableS1.pdf.

**Table S2:** ece371880‐sup‐0004‐TableS2.pdf.

**Data S1:** ece371880‐sup‐0005‐DataS1.pdf.

## Data Availability

All datasets are included in the paper's main text and appendix, as well as Supporting Information.

## Appendix 1 Covered Distances by Males and Females

**Table:**

Individual (*n* = 61)	Distance [m]	Individual (*n* = 29)	Distance [m]
1MA	27.7	1WA_F8	6.7
1MB	22	1WC_F24	24.8
1MC	48	1WD	28.1
1MD	16.7	1WE_F43	30.2
1ME_F2	14.2	1WF‐45WB_F25	64.5
1MF_F18	41.6	1WG	1.3
1MG_F36	35.4	1WH‐45WC	19.9
1MI	53.2	8WA	42.3
45MA‐1MJ_F44	90.6	13WA	1.2
45MB‐1MK_F51	86.7	6WB	27.2
19MA_F45	29.7	4WA_F13	40.0
19MC	20.7	7WC	24.7
8MB‐4MA‐5MB_F58	171.7	7WB	21.5
2MA_F1	29.9	4WB	17.3
2MB	10.3	12WA_F31	33.5
4MC	12.1	24WA	0.3
4MB_F68	18.3	15WD	3.1
4MD‐8MA_F7	103	15WA	29.1
4MH	55	15WB	11.1
4MJ	11	15WE	22.6
10MA	94.3	15WF	13.3
5MA‐4MF_F53	65.8	16WB‐15WJ	10.6
10MB	53.6	19WA_F46	28.4
4MG‐10MC	64.3	9WA_F57	13.1
10MD‐4ME‐5MC_8MC_F59	224.4	9WB	15.4
6MA‐13ME_F41	79.2	9WC	0.6
7MB‐6MC‐12MC	101.2	45WA	55.9
6MB	78.4	13WA	1.6
12MA‐7MA_F3	159.4	9WE	49.0
12MB‐24MI_F30	128.4		
13MC_F37	8.2		
13MD‐14MC_F38	152.7		
24MA‐13 MB‐14MB_F4	89.3		
24MB‐13MA‐6ME_F6	255.1		
24MC‐14MA_F32	100.1		
24MG‐14MD	32.8		
24MD	48.9		
24MH	53.1		
15MB_F15	31.9		
15MC_F19	3.4		
15MA‐16MA_F5	42.1		
15ME‐16MC_F52	120		
15MD‐16 MB_F20	16.7		
15MF	16.3		
15MH	70.9		
15MI	4.4		
15MJ	1.3		
15MK	43.8		
15ML	44.5		
15MN	45.7		
15MO‐16MF	176.8		
15MP	6.3		
15MQ‐16ME	15.2		
16MG	55		
9MA	70.9		
9MB	30.9		
9MC	13.7		
9MD	8.3		
9MF	41		
6MD	13		
24MF	48.9		

## Appendix 2 General Linear Model: Input (Males Distances, Age Class, Males and Females/50 m Radius & Activity Range, Sighting Number)

**Table:**

Individual male	Hatch year	Distance [m]	f/100 m	f/ar	m/100 m	m/ar	Sighting number
1MA	2015	27.7	3	2	4	3	5
1MB	2015	22	8	4	8	4	11
1MC	2014–2015	48	10	6	10	7	9
1MD	2014–2015	16.7	10	4	10	2	7
1ME_F2	2014	14.2	10	3	10	6	5
1MF_F18	2014	41.6	10	5	10	7	15
1MG_F36	2015	35.4	10	4	10	8	5
1MI	2014–2015	53.2	10	4	10	6	3
45MA‐1MJ_F44	2013	90.6	2	5	1	5	3
45MB‐1MK_F51	2014	86.7	2	5	1	5	8
19MA_F45	2013	29.7	2	2	2	2	3
19MC	2015	20.7	2	2	2	2	3
8MB‐4MA‐5MB_F58	2014–2015	171.7	6	6	10	10	12
2MA_F1	2014	29.9	2	1	3	3	11
2MB	2015	10.3	2	0	3	2	2
4MC	2015	12.1	4	1	11	6	2
4MB_F68	2014	18.3	6	2	10	7	3
4MD‐8MA_F7	2014	103	6	6	10	10	5
4MH	2014–2015	55	6	4	11	5	3
4MJ	2015	11	4	1	11	3	2
10MA	2014	94.3	2	3	2	3	5
5MA‐4MF_F53	2014	65.8	4	2	10	7	7
10MB	2014	53.6	5	2	8	3	4
4MG‐10MC	2014	64.3	5	3	11	9	6
10MD‐4ME‐5MC_8MC_F59	2015	224.4	6	7	10	12	10
6MA‐13ME_F41	2015	79.2	4	4	4	4	7
7MB‐6MC‐12MC	2014	101.2	4	3	2	3	6
6MB	2014–2015	78.4	4	4	4	4	3
12MA‐7MA_F3	2014	159.4	4	5	3	4	8
12MB‐24MI_F30	2014	128.4	2	2	2	4	4
13MC_F37	2015	8.2	3	0	4	2	3
13MD‐14MC_F38	2014–2015	152.7	3	3	6	4	5
24MA‐13 MB‐14MB_F4	2014	89.3	2	2	7	6	15
24MB‐13MA‐6ME_F6	2014	255.1	2	5	7	7	4
24MC‐14MA_F32	2014–2015	100.1	3	3	6	7	6
24MG‐14MD	2014	32.8	2	1	7	5	3
24MD	2015	48.9	0	0	1	0	5
24MH	2014–2015	53.1	2	1	7	5	8
15MB_F15	2013	31.9	6	3	8	5	9
15MC_F19	2014	3.4	5	2	5	5	4
15MA‐16MA_F5	2014	42.1	5	2	5	5	7
15ME‐16MC_F52	2013	120	1	3	1	7	4
15MD‐16MB_F20	2013	16.7	5	2	5	5	7
15MF	2014–2015	16.3	3	0	5	2	2
15MH	2014–2015	70.9	3	3	7	7	4
15MI	2014–2015	4.4	6	0	8	4	2
15MJ	2015	1.3	8	0	5	1	2
15MK	2014–2015	43.8	7	3	6	3	6
15ML	2014	44.5	7	3	6	3	4
15MN	2014	45.7	7	5	6	5	4
15MO‐16MF	2014–2015	176.8	3	8	6	14	4
15MP	2014–2015	6.3	7	0	6	4	3
15MQ‐16ME	2014–2015	15.2	5	2	5	5	2
16MG	2014–2015	55	3	2	4	1	2
9MA	2014–2015	70.9	3	3	4	4	5
9MB	2014–2015	30.9	3	2	4	4	3
9MC	2014–2015	13.7	3	2	4	4	2
9MD	2015	8.3	3	2	4	4	3
9MF	2015	41	3	2	4	4	4
6MD	2014–2015	13	4	0	4	4	2
24MF	2014–2015	48.9	2	2	7	5	2
