# Supplementary material for: Movement Patterns and Use of Habitat Corridors in Lacerta viridis in a Semi‐Natural Habitat
Source: Ecol Evol. 2025 Sep 11;15(9):e71880. doi: 10.1002/ece3.71880 (PMC12423634; doi:10.1002/ece3.71880)
Supplement: Supplementary file 1 — Figure S1: ece371880‐sup‐0001‐FigureS1.pdf. [file ECE3-15-e71880-s003.pdf]

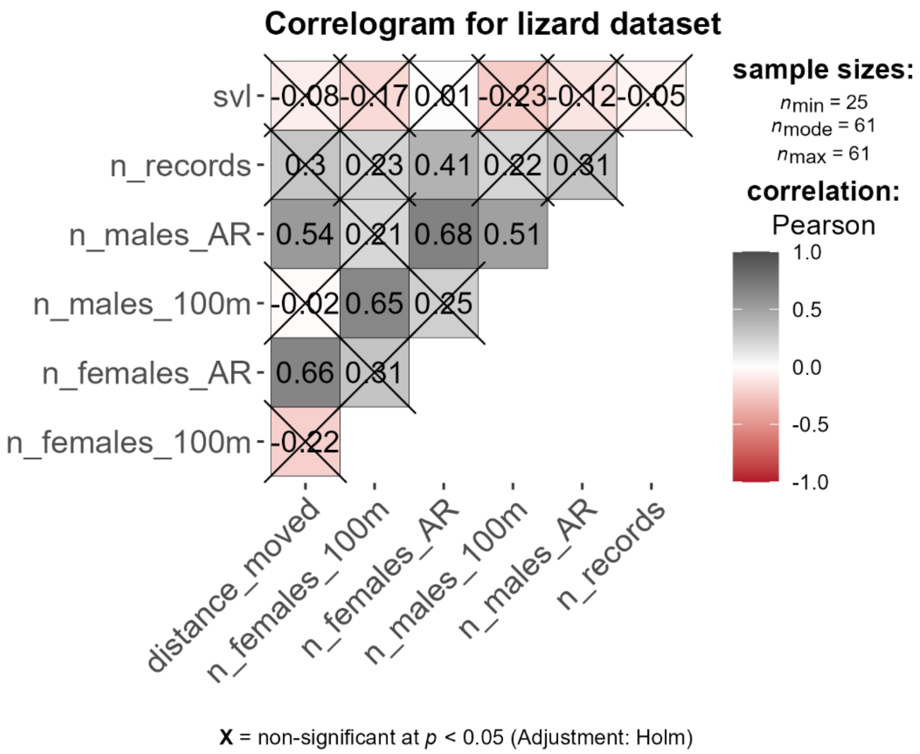

Figure S1: Correlogram illustrating pairwise correlations among key variables in the male lizard dataset. Each cell depicts the correlation coefficient (color-coded by strength and sign) between two variables, with darker shades indicating stronger correlations. The diagonal shows the distribution of each variable. The variables are as follows: distance_moved: Total distance (in meters) that an individual male lizard covered. n_females_100m: Number of female lizards within a male's home range. n_females_AR: Number of female lizards within the focal male's activity range. n_males_100m: Number of male lizards within a male's home range. n_males_AR: Number of male lizards within the focal male's activity range. n_records: Total number of records for each male lizard. svl: Snout–vent length, a standard measure of body size. Pearson correlation coefficients and p-values were computed using pairwise complete observations (Adjustment: Holm). Non-significant correlation pairs are represented by "X" in the respective cell.
