# Supplementary material for: Movement Patterns and Use of Habitat Corridors in Lacerta viridis in a Semi‐Natural Habitat
Source: Ecol Evol. 2025 Sep 11;15(9):e71880. doi: 10.1002/ece3.71880 (PMC12423634; doi:10.1002/ece3.71880)
Supplement: Supplementary file 2 — Figure S2: ece371880‐sup‐0002‐FigureS2.pdf. [file ECE3-15-e71880-s002.pdf]

Supplementary Figure S2

All sightings in habitats and corridors independent from number of individuals								
		**West**			**Middle part**			**East**
North	**Habitat sector**	**obj. 24**	**obj. 14**	**obj. 15**	**obj. 16**			**obj. 19**
	males	32	21	63	17			9
	females	2	2	24	2			6
	**Corridor**	**obj. 13**	**obj. 12**		**obj. 44**			**obj. 45**
	males	13	9		[4 subadults]			13
	females	6	7					7
South	**Habitat sector**	**obj. 7**	**obj. 6**	**obj. 5**	**obj. 4**	**obj. 3**	**obj. 2**	**obj. 1**
	males	14	14	4	40	4	15	76
	females	7	7	1	24	3	2	32
	**Corridor**			**obj. 10**	**obj. 8**			
	males			17	15			
	females			7	13			
