# Supplementary material for: Movement Patterns and Use of Habitat Corridors in Lacerta viridis in a Semi‐Natural Habitat
Source: Ecol Evol. 2025 Sep 11;15(9):e71880. doi: 10.1002/ece3.71880 (PMC12423634; doi:10.1002/ece3.71880)
Supplement: Supplementary file 3 — Table S1: ece371880‐sup‐0003‐TableS1.pdf. [file ECE3-15-e71880-s004.pdf]

Supplementary file S3

Tukey GLM results

contrast	estimate	SE	df	t.ratio	p.value
2014 - (2014-2015)	-0,026	0,178	53	-0,146	0,988
2014 - 2015	0,453	0,201	53	2,256	0,071
(2014-2015) - 2015	0,48	0,202	53	2,38	0,054

Post-hoc contrasts (Tukey-adjusted) suggested that pairwise differences in movement among hatch-year categories, while still showing trends for shorter distances covered in the 2015 cohort compared to both other groups, did not remain significant at the $\alpha = 0.05$ level (all $p > 0.05$), likely reflecting reduced power after multiple-comparison adjustments.
