# Supplementary material for: Movement Patterns and Use of Habitat Corridors in Lacerta viridis in a Semi‐Natural Habitat
Source: Ecol Evol. 2025 Sep 11;15(9):e71880. doi: 10.1002/ece3.71880 (PMC12423634; doi:10.1002/ece3.71880)
Supplement: Supplementary file 4 — Table S2: ece371880‐sup‐0004‐TableS2.pdf. [file ECE3-15-e71880-s005.pdf]

Supplementary file S4

Tukey SVL

contrast	estimate	SE	df	t.ratio	p.value
2014 - (2014-2015)	0,978	0,381	22	2,563	0,045
2014 - 2015	1,457	0,338	22	4,309	0,001
(2014-2015) - 2015	0,479	0,467	22	1,026	0,569

Post-hoc tests (Tukey-adjusted) indicating that males from 2014 were larger than those from both 2014–2015 ($p = 0.045$, Supplementary Table S4) and 2015 ($p < 0.001$), whereas these latter two groups did not differ from each other ($p=0.57$).
