# Supplementary material for: Movement Patterns and Use of Habitat Corridors in Lacerta viridis in a Semi‐Natural Habitat
Source: Ecol Evol. 2025 Sep 11;15(9):e71880. doi: 10.1002/ece3.71880 (PMC12423634; doi:10.1002/ece3.71880)
Supplement: Supplementary file 5 — Data S1: ece371880‐sup‐0005‐DataS1.pdf. [file ECE3-15-e71880-s001.pdf]

Supplementary file S5

R-Scripts for analyses

Movement patterns and use of habitat corridors in *Lacerta viridis* in a semi-natural habitat

```
---
title: "Lizards at Nussberg"
author: "Victor Scharnhorst"
date: "`r Sys.Date()`"
output: html_notebook
---


# Setup
load packages and set wd
hallo das ist das eidechsen skript
```{r setup, include=TRUE}
rm(list=ls()) #deletes your current environment, deactivate if not needed
getwd()
library(tidyverse)
library(rio)
library(MASS)
library(car)
library(viridis)
library(ggstatsplot)
library(vegan)
library(lme4)
library(knitr)
library(emmeans)
library(DHARMa)
library(ggpubr)
library(ggsci)
library(performance)
library(emmeans)
```
```

````
library(broom)

library(openxlsx)


knitr::opts_chunk$set(

  echo = FALSE,         # Show the R code in the output document

  warning = FALSE,      # Do not display warnings

  message = FALSE,      # Do not display messages

  fig.width = 6,        # Set the default figure width

  fig.height = 4.5,     # Set the default figure height

  cache = FALSE         # Do not cache results by default

)


current_date <- Sys.Date()

formatted_date <- format(current_date, "%Y%m%d")


```


# Load data


```{r}

lizards_raw <- rio::import("lacerta_raw_data.xlsx", stringsAsFactors = T, sheet = 1)   # convenience function from package "rio"


lizards_raw_SVL <- rio::import("lacerta_raw_data.xlsx", stringsAsFactors = T, sheet = 3)   # convenience function from package "rio"


lizards_raw_fm <- rio::import("lacerta_raw_data.xlsx", stringsAsFactors = T, sheet = 2)


lizards_prep_fm <- lizards_raw_fm %>%

  dplyr::select(Individuum, `Distanz Female`) %>%

  rename(distance_moved = `Distanz Female`,

         ID = Individuum) %>%
````

```
  drop_na(ID) %>%
  mutate(sex = "female") %>%
  full_join(lizards_raw_fm %>%
            dplyr::select(`Individual male`, Distanz) %>%
  rename(distance_moved = Distanz,
      ID = `Individual male`) %>%
  drop_na(ID) %>%
  mutate(sex = "male")
  )


temp <- lizards_raw_SVL %>%
   rename(
   male_ID = Individuum,
   hatch_year = Jahrgang,
   distance_moved = Distanz,
   n_females_100m = `w/100m*`,
   n_females_AR = `w/Aktionsradius`,
   n_males_100m = `m/100m*`,
   n_males_AR = `m/Aktionsradius`,
   n_records = `Sichtungen`,
   svl = SVL
  )

lizards_prep <- lizards_raw %>%
  rename(
   male_ID = `Individual male`,
   hatch_year = `Hatch year`,
   distance_moved = `Distance [m]`,
   n_females_100m = `f/100m`,
   n_females_AR = `f/ar`,
   n_males_100m = `m/100m`,
```

```
    n_males_AR = `m/ar`,
    n_records = `Sichtungen Anzahl`
  ) %>%
  full_join(temp %>%  dplyr::select(male_ID, svl)) %>%
  mutate(hatch_year = as.factor(hatch_year),
         age_male = case_when(
         hatch_year == "2014" ~ "3",
         hatch_year == "2015" ~ "2",
         hatch_year == "2014-2015" ~ "2.5"
         ), # Age of lizards is calculated as 2017 - hatch_year
         age_male = as.numeric(age_male)
         ) %>%
  mutate(hatch_year = case_when(
    hatch_year == "2013" ~ "2014",
    TRUE ~ hatch_year),
    hatch_year = factor(hatch_year, levels = c("2014", "2014-2015", "2015")))
```

Analysis

comparison males and females

```{r}

# Histogram of the response variable: distance_moved
hist(lizards_prep_fm$distance_moved, breaks = 15,
     main = "Histogram of Distance Moved",
     xlab = "Distance Moved (m)",
     col = "skyblue")

# Histogram of the response variable: distance_moved
hist(log(lizards_prep_fm$distance_moved), breaks = 15,
     main = "Histogram of log(Distance Moved)",
```

```
    xlab = "Distance Moved log(m)",
    col = "skyblue")

glimpse(lizards_prep_fm %>% arrange(distance_moved))

p <- lizards_prep_fm %>%
  mutate(distance_moved = log(distance_moved)) %>%
  ggbetweenstats(
  x     = sex,
  y     = distance_moved,
  #title = "Distribution of distance moved by individuals across sex",
  type = "parametric",
  results.subtitle = F,
  xlab = "Lizard Sex",
  ylab = "log(Distance Covered)"
)
p
ggsave(paste0("Lacerta_fm_boxplot_", formatted_date, ".png"), p, width = 6, height = 4, dpi = 300)

aov_fm <- aov(log(distance_moved) ~ sex, data= lizards_prep_fm)

summary(aov_fm)

str(lizards_prep_fm)
str(lizards_prep)

library(dplyr)

# 1. Summary of distances moved by sex
distance_summary <- bind_rows(
  # males: from lizards_prep
  lizards_prep %>%
```

```
    summarise(
      sex           = "male",
      mean_distance  = mean(distance_moved, na.rm = TRUE),
      sd_distance    = sd(distance_moved, na.rm = TRUE),
      n_individuals  = n()
    ),
  # females: from lizards_prep_fm
  lizards_prep_fm %>%
    filter(sex == "female") %>%
    summarise(
      sex           = "female",
      mean_distance  = mean(distance_moved, na.rm = TRUE),
      sd_distance    = sd(distance_moved, na.rm = TRUE),
      n_individuals  = n()
    )
)

# 2. Raw count of sightings per male
male_records <- lizards_prep %>%
  dplyr::select(n_records, male_ID) %>%
  summarise(
      mean_sightings  = mean(n_records, na.rm = TRUE),
      sd_records    = sd(n_records, na.rm = TRUE)
  ) %>%
  mutate(sex = "male")

# 3. Raw counts of sightings per female
female_records <- import("Tabelle_Sichtungshäufigkeiten M_&_ F.xlsx", sheet = 2) %>%
  rename(distance = Distance,
         n_records = `Record number`) %>%
    summarise(
      mean_sightings  = mean(n_records, na.rm = TRUE),
```

```
    sd_records    = sd(n_records, na.rm = TRUE)
  ) %>%
  mutate(sex = "female")

summary_table <- full_join(male_records, female_records) %>%
  full_join(distance_summary) %>%
  relocate(sex, n_individuals)
# Inspect
distance_summary
male_records
str(summary_table)

# 1. Round and rename columns for clarity
summary_export <- summary_table %>%
  mutate(across(where(is.numeric), ~ round(., 3))) %>%
  rename(
    Sex                  = sex,
    `Mean Sightings (n)`    = mean_sightings,
    `SD Sightings (n)`      = sd_records,
    `Mean Distance Moved (m)` = mean_distance,
    `SD Distance Moved (m)`   = sd_distance,
    `Number of Individuals` = n_individuals
  )

# 2. Export to CSV
write.csv(
  summary_export,
  file = "summary_table.csv",
  row.names = FALSE
)

library(officer)
```

````
library(flextable)

# 3. Create a Word document with a legend and the table

doc <- read_docx()


# Add a heading for the table

doc <- body_add_par(doc, "Table 1. Summary of Lizards’ Movement and Sightings", style = "heading 2")


# Add a legend describing the columns

doc <- body_add_par(

  doc,

  "Legend: Mean ± SD of distances moved (in meters) by sex; and mean ± SD of the number of sightings per individual (males only).",

  style = "Normal"

)


# Turn the summary_export data.frame into a flextable and auto-fit

ft <- flextable(summary_export) %>%

  autofit()


# Add the flextable to the document

doc <- body_add_flextable(doc, value = ft)

print(doc, target = paste0("summary_table", formatted_date, ".docx"))


```




## correlation of predictors and data structure

```{r}

library(Hmisc)    # For correlation significance (rcorr)


# --- Summary Table by Age Class ---
````

```
# This table provides mean and standard deviation for distance moved,
# and the mean number of females in the home (100m) and activity ranges.
summary_table <- lizards_prep %>%
  group_by(hatch_year) %>%
  summarise(
    mean_distance = mean(distance_moved, na.rm = TRUE),
    sd_distance = sd(distance_moved, na.rm = TRUE),
    mean_n_females_100m = mean(n_females_100m, na.rm = TRUE),
    mean_n_females_AR = mean(n_females_AR, na.rm = TRUE)
  )

# Print the summary table
print(summary_table)

p <- lizards_prep %>%
  ggbetweenstats(
  x     = hatch_year,
  y     = distance_moved,
  title = "Distribution of distance moved by male individuals across age"
)
p



# --- One-Way ANOVA ---
# Test whether distance moved differs among the three age classes.
anova_result <- aov(distance_moved ~ hatch_year, data = lizards_prep)
print(summary(anova_result))

# --- Correlation Analysis ---
# Select continuous variables for correlation analysis.
num_vars <- lizards_prep %>%
```

````
  dplyr::select(distance_moved, n_females_100m, n_females_AR, n_males_100m, n_males_AR,
n_records, svl)

# Compute the correlation matrix using pairwise complete observations.
cor_matrix <- cor(num_vars, use = "pairwise.complete.obs")
print(cor_matrix)

# Optionally, use the Hmisc package to obtain correlation coefficients along with significance levels.
cor_results <- rcorr(as.matrix(num_vars))
print(cor_results)  # Contains correlation coefficients, n (sample size) and p-values

p <- num_vars %>%
  ggcorrmat(
  colors   = c("#B2182B", "white", "#4D4D4D"),
  title    = "Correlogram for lizard dataset"#,
  #subtitle = "distance moved by males, number of females in 100m radius, number of f in action
radius of m, number of m in 100m, number of m in ar"
)
p

ggsave(paste0("Lacerta_corrplot_GLM_", formatted_date, ".png"), p, width = 6, height = 4, dpi = 300)

```

## GLM
```{r}
lizards_mod <- lizards_prep
str(lizards_mod)

# check for correlation among predictors
# Examine the overall structure of the data frame
str(lizards_mod)
````

```
# Get summary statistics for all variables
summary(lizards_mod)

# Check for missing values in each column
sapply(lizards_mod, function(x) sum(is.na(x)))

# ----- Visualizing Distributions -----

# Histogram of the response variable: distance_moved
hist(lizards_mod$distance_moved, breaks = 15,
    main = "Histogram of Distance Moved",
    xlab = "Distance Moved (m)",
    col = "skyblue")

# Histogram of the response variable: distance_moved
hist(log(lizards_mod$distance_moved), breaks = 15,
    main = "Histogram of log(Distance Moved)",
    xlab = "Distance Moved log(m)",
    col = "skyblue")


# ----- Visualizing Relationships -----

# Scatterplot: Distance Moved vs Age of Male
plot(lizards_mod$age_male, lizards_mod$distance_moved,
    xlab = "Age of Male", ylab = "Distance Moved (m)",
    main = "Distance Moved vs Age of Male")

# Scatterplot: Distance Moved vs Number of Females (100m)
plot(lizards_mod$n_females_100m, lizards_mod$distance_moved,
    xlab = "Number of Females (100m)", ylab = "Distance Moved (m)",
```

```r
    main = "Distance Moved vs Number of Females (100m)")

# Scatterplot: Distance Moved vs Number of Males (100m)
plot(lizards_mod$n_males_100m, lizards_mod$distance_moved,
    xlab = "Number of Males (100m)", ylab = "Distance Moved (m)",
    main = "Distance Moved vs Number of Males (100m)")

# A scatterplot matrix for a quick overview of relationships among key variables
pairs(lizards_mod[, c("distance_moved", "age_male", "n_females_100m", "n_males_100m")],
     main = "Scatterplot Matrix")

# Boxplot: Compare Distance Moved by Hatch Year groups
boxplot(distance_moved ~ hatch_year, data = lizards_mod,
       main = "Distance Moved by Hatch Year",
       xlab = "Hatch Year", ylab = "Distance Moved (m)")

# GLM



glm_mod <- glm(log(distance_moved) ~ # log transformation to normalize data
              hatch_year +
              n_females_100m +
              n_females_AR +
              n_males_100m +
              n_males_AR +
              n_records,
             data = lizards_mod)

summary(glm_mod)
check_collinearity(glm_mod)
sim_res <- simulateResiduals(fittedModel = glm_mod, n = 1000)
plot(sim_res)                # visually check residual patterns
```

```r
testDispersion(sim_res)      # over-/underdispersion
testZeroInflation(sim_res)   # zero inflation
testUniformity(sim_res)

glm_tidy <- tidy(glm_mod) %>%
  mutate(across(
    where(is.numeric),
    ~ {
      val <- round(.x, 3)
      # If the rounded value is 0, replace with "<0.001", otherwise convert the number to string
      ifelse(val == 0, "<0.001", as.character(val))
    }
  ))
str(glm_tidy)
# 'glm_tidy' contains columns for term (the predictor), estimate, std.error, statistic, p.value, etc.

# 2. Export the GLM results to a CSV file
write.xlsx(glm_tidy, file = paste0("glm_results_", formatted_date, ".xlsx"), row.names = FALSE)

# Estimate marginal means for hatch_year
emm <- emmeans(glm_mod, ~ hatch_year)

# Perform pairwise comparisons with Tukey adjustment
posthoc_results <- pairs(emm, adjust = "tukey")

# Print the summary of the comparisons
summary(posthoc_results)

tukey_tidy <- as.data.frame(summary(posthoc_results)) %>%
  mutate(across(where(is.numeric), ~ round(.x, 3)))
# 'tukey_tidy' will contain columns like: contrast, estimate, SE, df, t.ratio, p.value
```

```
write.xlsx(tukey_tidy, file = paste0("tukey_glm_results_", formatted_date, ".xlsx"), row.names =
FALSE)


# install.packages("MuMIn")  # if you haven't already

library(MuMIn)


# 1. Make sure your global model uses NA-fail so dredge considers all subsets

options(na.action = "na.fail")


# 3. Run dredge to get all possible submodels, ranked by AICc

dredge_res <- dredge(glm_mod,

                  rank     = "AICc",

                  trace    = TRUE,     # show progress

                  fixed    = NULL)     # no terms forced in all models


# 4. Inspect the top models (delta AICc < 2)

top_models <- subset(dredge_res, delta < 2)

print(top_models)


# 4.1 Compare Top Model 40 with selected Model 64

mod_40 <- glm(log(distance_moved) ~ # log transformation to normalize data

              hatch_year +

              n_females_100m +

              n_females_AR +

              #n_males_100m +

              #n_males_AR +

              n_records,

            data = lizards_mod)


anova(glm_mod, mod_40) # no sign difference


# 5. (Optional) Model-averaging over the top set
```

avg_mod <- model.avg(top_models)

summary(avg_mod)

6. Reset na.action if needed

options(na.action = "na.omit")

```

### export top model table

```{r}

library(officer)

library(flextable)

# 3. Create a Word document with a legend and the table

doc <- read_docx()

# Add a heading for the table

doc <- body_add_par(doc, "Table XXX. GLM candidate models and their AICc values based on model selection procedure", style = "heading 1")

# Add a legend describing the columns

doc <- body_add_par(

  doc,

  "Model selection results for competing generalized linear models (GLM) predicting log-transformed male movement distance. Each row shows a candidate model (by model ID), its intercept ((Int)), which predictors are included (“+” indicates inclusion), the estimated coefficients for female counts in the 100 m home range (n_fml_100) and activity range (n_fml_AR), male counts in the 100 m home range (n_mls_100) and activity range (n_mls_AR), and sampling effort (n_rcr). The remaining columns give the model’s degrees of freedom (df), log-likelihood (logLik), small-sample corrected AIC (AICc), ΔAICc (difference from the best model), and Akaike weight (relative support).",

  style = "Normal"

)

# Turn the summary_export data.frame into a flextable and auto-fit

ft <- as.data.frame(top_models) %>%

  mutate(

~~~
    across(
      .cols = where(is.numeric),
      .fns = ~ round(.x, 3)
    )
  ) %>%
  flextable() %>%
  autofit()

# Add the flextable to the document
doc <- body_add_flextable(doc, value = ft)
print(doc, target = paste0("candidate_models_table", formatted_date, ".docx"))
```


glm and tests for svl data
```{r}

lizards_mod <- lizards_prep %>%
  filter(!is.na(svl))
str(lizards_mod)

p <- lizards_mod %>%
  ggbetweenstats(
  x     = hatch_year,
  y     = svl,
  title = "Distribution of SVL by male individuals across age"
)
p

p <- lizards_mod %>%
  ggscatterstats(
  x     = distance_moved,
~~~

```
  y     = svl,
  title = "Distribution of distance moved by male individuals across SVL"
)
p

# Histogram of the response variable: distance_moved
hist(lizards_mod$svl, breaks = 15,
     main = "Histogram of SVL of males",
     xlab = "SVL",
     col = "skyblue")

shapiro.test(lizards_mod$svl)

glm_mod <- glm(log(distance_moved) ~ # log transformation to normalize data
                 svl +
                 #hatch_year +
                 #n_females_100m +
                 #n_females_AR +
                 #n_males_100m +
                 #n_males_AR +
                 n_records,
               #family = Gamma(link = "log"),
               data = lizards_mod)

summary(glm_mod)
check_collinearity(glm_mod)
sim_res <- simulateResiduals(fittedModel = glm_mod, n = 1000)
plot(sim_res)                  # visually check residual patterns
testDispersion(sim_res)       # over-/underdispersion
testZeroInflation(sim_res)    # zero inflation
testUniformity(sim_res)
```

```
aov_svl <- aov(log(distance_moved) ~ svl, data= lizards_mod)

aov_hatch <- aov(svl ~ hatch_year, data= lizards_mod)

summary(aov_svl)

summary(aov_hatch)


# Estimate marginal means for hatch_year

emm <- emmeans(aov_hatch, ~ hatch_year)


# Perform pairwise comparisons with Tukey adjustment

posthoc_results <- pairs(emm, adjust = "tukey")


# Print the summary of the comparisons

summary(posthoc_results)


tukey_tidy <- as.data.frame(summary(posthoc_results)) %>%

  mutate(across(where(is.numeric), ~ round(.x, 3)))

# 'tukey_tidy' will contain columns like: contrast, estimate, SE, df, t.ratio, p.value

write.xlsx(tukey_tidy, file = paste0("tukey_aov_results_", formatted_date, ".xlsx"), row.names = FALSE)
```
